# Supplementary material for: EnzML: multi-label prediction of enzyme classes using InterPro signatures
Source: BMC Bioinformatics. 2012 Apr 25;13:61. doi: 10.1186/1471-2105-13-61 (PMC3483700; doi:10.1186/1471-2105-13-61)
Supplement: Addtional file 5 — The Java code to format the data files, evaluate and predict. The file enzml_java_code.tar.gz contains the Java code used to format database data to ARFF and XML formats, to execute cross and train-test (jackknife) evaluations and to record evaluation results to database. More information is included in the readme.txt file and the Javadoc files. The code can be used with a MySQL database. To use a different database software, other JDBC drivers might be required. [file 1471-2105-13-61-S5.gz › java_code/enzml2011/doc/uk/ac/ed/inf/taweka/mulan/MulanAttributeFactory.html]

MulanAttributeFactory


---


|  |  |  |
| --- | --- | --- |
| **Overview** \| **Package** \| **Class** \| **Use** \| **Tree** \| **Deprecated** \| **Index** \| **Help** | |  |
| **PREV CLASS**   **NEXT CLASS** | **FRAMES**    **NO FRAMES**     **All Classes** |  |
| SUMMARY: NESTED \| FIELD \| CONSTR \| METHOD | DETAIL: FIELD \| CONSTR \| METHOD |  |


---


## uk.ac.ed.inf.taweka.mulan Class MulanAttributeFactory

```
java.lang.Object
  uk.ac.ed.inf.taweka.weka.AttributeFactory
      uk.ac.ed.inf.taweka.mulan.MulanAttributeFactory
```

**Direct Known Subclasses:**: AttributesFilteredAttributeFactory

---

``` public class MulanAttributeFactory extends AttributeFactory ```

Class

**Version:**
:   7 May 2010

**Author:**
:   Luna De Ferrari luna.deferrari-at-ed.ac.uk

---

| **Field Summary** | |
| --- | --- |
| `static double` | `ABSENT_OPTION` |
| `static double` | `PRESENT_OPTION` |


**Constructor Summary**

`MulanAttributeFactory(MulanDataSetGenerator dataSetGenerator)`


| **Method Summary** | |
| --- | --- |
| `weka.core.Attribute` | `generateBinaryAttribute(java.lang.String attributeName)`             Generates a binary attribute |
| `java.lang.String` | `getAttributeAbsentOption()` |
| `java.lang.String` | `getAttributePresentOption()` |
| `java.util.Vector<java.lang.String>` | `getBinaryAttributeValuesSet()`             Get the possible attribute values for a yes/no attribute. |
| `MulanDataSetGenerator` | `getDataSetGenerator()` |

**Methods inherited from class uk.ac.ed.inf.taweka.weka.AttributeFactory**

`generateNominalAttribute, generateNominalAttributeWithDummy, getAttributeByName, getAttributes, getAttributesNames, getClassAttribute`

**Methods inherited from class java.lang.Object**

`equals, getClass, hashCode, notify, notifyAll, toString, wait, wait, wait`

**Field Detail**

### ABSENT\_OPTION

```
public static final double ABSENT_OPTION
```

**See Also:**: Constant Field Values

---


### PRESENT\_OPTION

```
public static final double PRESENT_OPTION
```

**See Also:**: Constant Field Values


**Constructor Detail**

### MulanAttributeFactory

```
public MulanAttributeFactory(MulanDataSetGenerator dataSetGenerator)
```


**Method Detail**

### generateBinaryAttribute

```
public weka.core.Attribute generateBinaryAttribute(java.lang.String attributeName)
```

:   **Description copied from class: `AttributeFactory`**
:   Generates a binary attribute

    :   **Overrides:**: `generateBinaryAttribute` in class `AttributeFactory`
    :   **Parameters:**: `attributeName` - the attribute name **Returns:**: the weka attribute

---


### getAttributeAbsentOption

```
public java.lang.String getAttributeAbsentOption()
```

:   **Overrides:**: `getAttributeAbsentOption` in class `AttributeFactory`

---


### getAttributePresentOption

```
public java.lang.String getAttributePresentOption()
```

:   **Overrides:**: `getAttributePresentOption` in class `AttributeFactory`

---


### getBinaryAttributeValuesSet

```
public java.util.Vector<java.lang.String> getBinaryAttributeValuesSet()
```

:   **Description copied from class: `AttributeFactory`**
:   Get the possible attribute values for a yes/no attribute. Beware!: the
    option "no" has to be listed as first option for the sparse ARFF format
    to work! http://weka.wikispaces.com/ARFF+%28stable+version%29

    :   **Overrides:**: `getBinaryAttributeValuesSet` in class `AttributeFactory`
    :   **Returns:**: The vector with the two values, for example: ["no", "yes"].

---


### getDataSetGenerator

```
public MulanDataSetGenerator getDataSetGenerator()
```

:   **Overrides:**: `getDataSetGenerator` in class `AttributeFactory`


---


|  |  |  |
| --- | --- | --- |
| **Overview** \| **Package** \| **Class** \| **Use** \| **Tree** \| **Deprecated** \| **Index** \| **Help** | |  |
| **PREV CLASS**   **NEXT CLASS** | **FRAMES**    **NO FRAMES**     **All Classes** |  |
| SUMMARY: NESTED \| FIELD \| CONSTR \| METHOD | DETAIL: FIELD \| CONSTR \| METHOD |  |


---
